# Stathmin 1 inhibition amplifies ruxolitinib-induced apoptosis in JAK2^V617F^ cells

**DOI:** 10.18632/oncotarget.4998

**Published:** 2015-08-17

**Authors:** João Agostinho Machado-Neto, Paula de Melo Campos, Patricia Favaro, Mariana Lazarini, Adriana da Silva Santos Duarte, Irene Lorand-Metze, Fernando Ferreira Costa, Sara Teresinha Olalla Saad, Fabiola Traina

**Affiliations:** ^1^ Hematology and Hemotherapy Center, University of Campinas/Hemocentro-Unicamp, Instituto Nacional de Ciência e Tecnologia do Sangue, Campinas, São Paulo, Brazil; ^2^ Current address: Department of Biological Sciences, Federal University of São Paulo, Diadema, São Paulo, Brazil; ^3^ Current address: Department of Internal Medicine, University of São Paulo at Ribeirão Preto Medical School, Ribeirão Preto, São Paulo, Brazil

**Keywords:** myeloproliferative neoplasms, STAT3, stathmin 1, ruxolitinib, paclitaxel

## Abstract

The JAK/STAT pathway is constitutively activated in myeloproliferative neoplasms and can be inhibited by ruxolitinib, a selective JAK1/2 inhibitor. The JAK2^V617F^ mutation leads to constitutive STAT3 phosphorylation and potentially leads to inhibition of Stathmin 1 activity via STAT3. In support of this hypothesis, we found that, in HEL JAK2^V617F^ cells, ruxolitinib treatment decreased STAT3 and Stathmin 1 association, induced Stathmin 1 activation and microtubule instability. Silencing of Stathmin 1 significantly reduced cell proliferation and clonal growth, and increased apoptosis induced by ruxolitinib. Stathmin 1 silencing also prevented ruxolitinib-induced microtubule instability. To phenocopy the effect of Stathmin 1 inhibition, cells were treated with paclitaxel, a microtubule-stabilizing drug, in association or not with ruxolitinib; combined treatment significantly increased apoptosis, when compared to monotherapy. Notably, *Stathmin 1* mRNA levels were highly expressed in CD34^+^ cells from primary myelofibrosis patients. We then proposed that an undesired effect of ruxolitinib treatment may constitute Stathmin 1 activation and microtubule instability in JAK2^V617F^ cells. Induction of microtubule stability, through Stathmin 1 silencing or paclitaxel treatment, combined with ruxolitinib could be an effective strategy for promoting apoptosis in JAK2^V617F^ cells.

## INTRODUCTION

Philadelphia chromosome-negative myeloproliferative neoplasms (MPNs), including essential thrombocythemia (ET), polycythemia vera (PV) and primary myelofibrosis (PMF), are characterized by increased myeloid proliferation, with predominant megakaryocytic, erythroid, and megakaryocytic/granulocytic expansion, respectively, and have a potential to leukemia transformation [1]. A gain of function mutation, V617F, in *Janus kinase 2* (*JAK2*) gene has been reported in most PV cases and in more than half of ET and PMF cases [2]. However, the presence of multiple disease phenotypes and the absence of JAK2 mutation in some cases of MPN indicates that additional genetic lesions or/and aberrant signaling pathways may be involved in the pathogenesis and progression of the MPN [1, 2].

Stathmin 1, also named Oncoprotein 18 (OP18) or Leukemia-associated phosphoprotein p18 (LAP18), is a microtubule destabilizer that plays an important function in cell proliferation, clonogenicity, differentiation, motility and survival [3]. In normal hematopoiesis, high Stathmin 1 expression correlates with the proliferative ability of early hematopoietic progenitors, and the downregulation of this protein is required for efficient cell differentiation [4, 5]. Stathmin 1 knockout mice presented two human-like phenotypes of hematopoietic disorders; megaloblastic anemia and thrombocytosis [6]. In malignant hematopoiesis, Stathmin 1 overexpression was reported in acute myeloid leukemia, acute lymphoid leukemia and myelodysplastic syndromes [7, 8, 9]. Notably, *Stathmin 1* silencing reduces cell proliferation and clonogenicity of leukemia cell lines [9, 10, 11].

Signaling pathways deregulated in MPN have the potential to regulate Stathmin 1 activity. For instance, PI3K, ERK1/2 and JNK1/2 regulate the activity of Stathmin 1 through its phosphorylation at serine 25 and/or 38 [3, 12]. More importantly, activated STAT3 binds to and inhibits Stathmin 1, resulting in microtubule stability in non-Hodgkin lymphoma and gastric cancer human cell lines [13, 14], but Stathmin 1 has never been investigated in MPN. Thus, we aimed to investigate, in a JAK2^V617F^ cell line, Stathmin 1 function and the effects of ruxolitinib on Stathmin 1 activation and cell phenotype. We also aimed to evaluate Stathmin 1 expression in CD34^+^ cells from BCR-ABL1 negative MPN patients.

## RESULTS

### Ruxolitinib treatment increases microtubule instability

**Figure 1:**
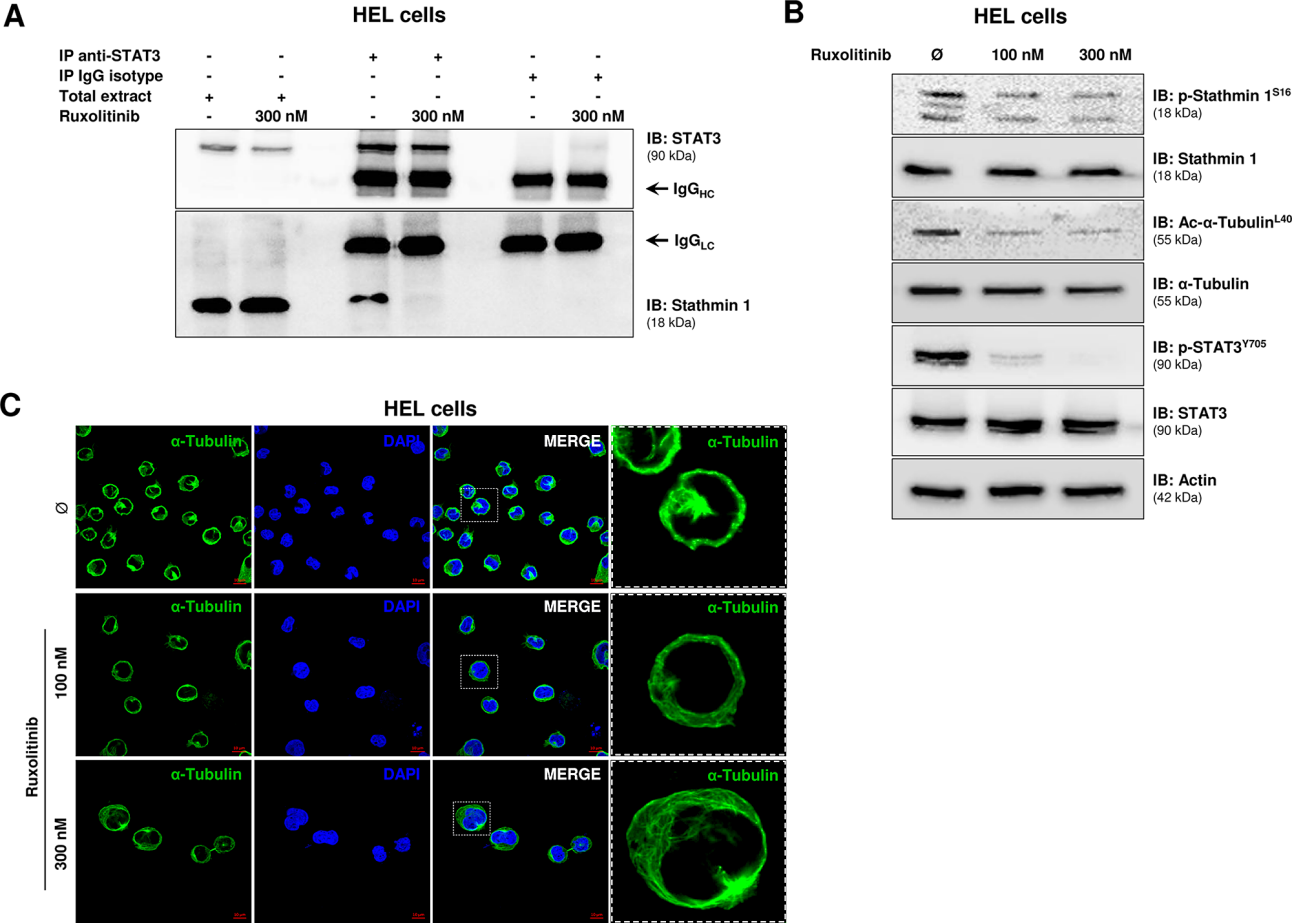
Ruxolitinib treatment induces microtubule instability **A.** Immunoprecipitation (IP) with anti-STAT3 and immunobloting (IB) with anti-Stathmin 1 in total extracts from HEL cells, treated or not with ruxolitinib (300 nM). Isotype IgG antibody was used as a negative control of the immunoprecipitation; total cell extracts were used as positive controls for immunoblotting. **B.** Western blot analysis for Stathmin 1 serine 16 phosphorylation (an inhibitory site) (p-Stathmin 1^S16^) and alpha-tubulin acetylation (a marker of microtubule stability) (Ac-α-Tubulin^L40^) levels in total cell extracts from HEL cells treated or not with different concentration of ruxolitinib (100 and 300 nM). The antibodies used for immunoblotting (IB) are indicated. The decreased p-Stathmin 1 and Ac-α-Tubulin levels indicate Stathmin 1 activation and microtubule instability upon ruxolitinib treatment. Immunoblotting for phospho STAT3 and total STAT3 confirmed the inhibitory effect of ruxolitinib on STAT3 activation; actin was used as a loading control. **C.** Confocal analysis of HEL cells, treated or not with different concentration of ruxolitinib (100 and 300 nM), displaying α-Tubulin (green) and DAPI (blue) staining; MERGE shows the overlapped images. Scale bars are shown in the figure (10 μm). Note more diffuse microtubule networks in ruxolutinib-treated cells.

Given that STAT3 binds to and inhibits Stathmin 1 in non-Hodgkin lymphoma and gastric cancer human cell lines [13, 14], we first confirmed the association of STAT3 and Stathmin 1 in HEL cells, which was abrogated by ruxolitinib treatment (Figure 1A), possibly due to STAT3 phosphorylation inhibition induced by ruxolitinib [15]. Next, we evaluated the effects of ruxolitinib treatment on Stathmin 1 activity and microtubule stability by assessment of Stathmin 1 serine 16 phosphorylation (an inhibitory site), alpha-tubulin acetylation (a marker of microtubule stability) and confocal analysis of microtubule networks in the JAK2^V617F^ cell model. In HEL cells, ruxolitinib treatment induced a slight decrease in Stathmin 1 phosphorylation and a marked reduction of alpha-tubulin acetylation, indicating increased microtubule instability (Figure 1B). Confocal analysis corroborated our results obtained with the microtubule stability markers, evidencing that ruxolitinib-treated HEL cells present a more diffuse microtubule network (Figure 1C).

### Stathmin 1 silencing reduces cell proliferation and clonogenicity, and increases the pro-apoptotic effects of ruxolitinib

**Figure 2:**
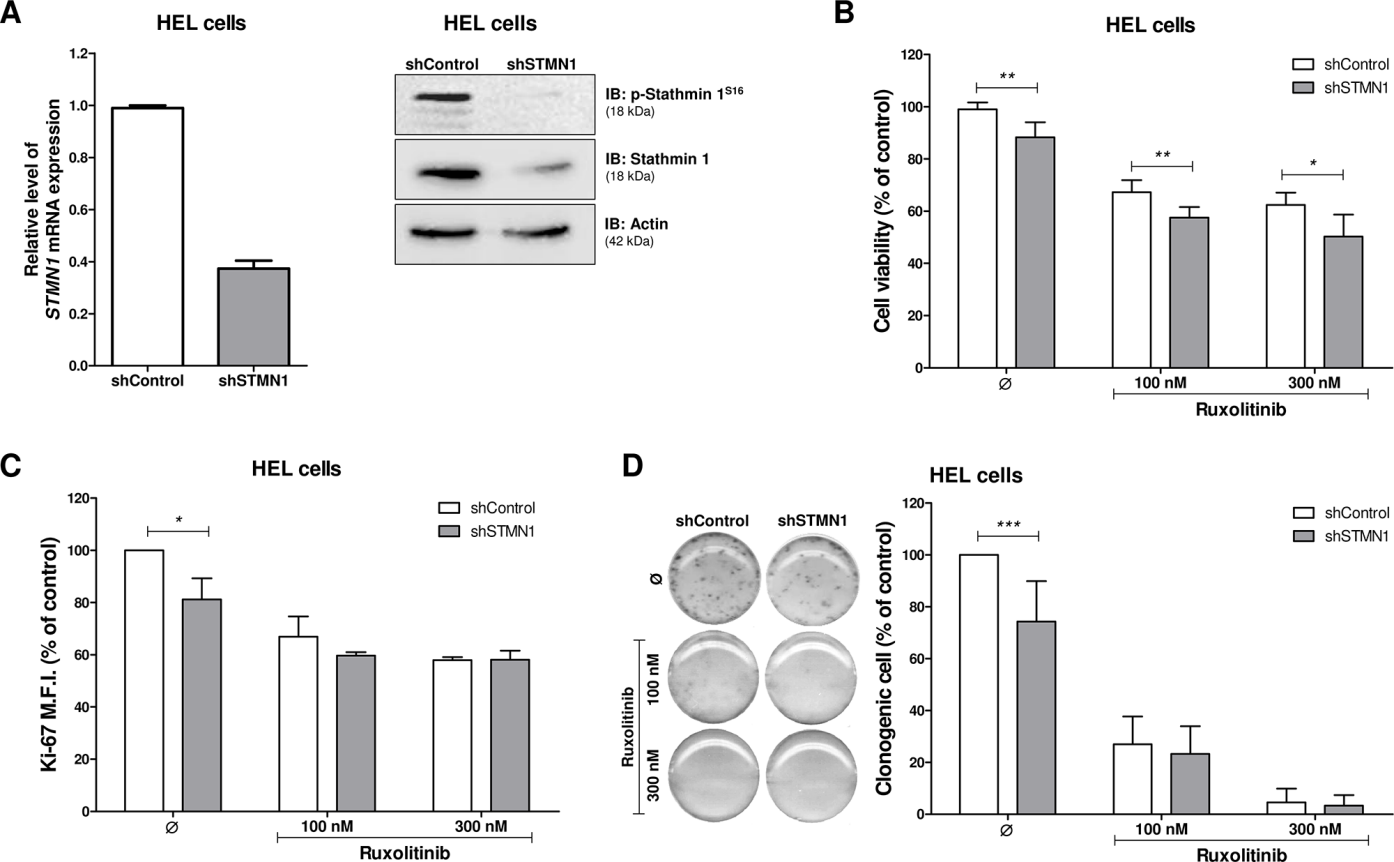
Stathmin 1 silencing reduces cell proliferation and clonogenicity **A.** Stathmin 1 mRNA and protein expression in HEL cells transduced with lentivirus-mediated shRNA control (shControl) or Stathmin 1 (shSTMN1). The antibodies used for immunoblotting (IB) are indicated. **B.** Cell viability was determined by MTT assay after 48 hours of incubation of shSTMN1 and normalized by the corresponding shControl cells. Results are shown as mean ± SD of six independent experiments; *p < 0.05, **p < 0.01; *Mann–Whitney* test. **C.** Ki-67 mean of fluorescence intensity (M.F.I.) was determinated by flow cytrometry after incubation of shSTMN1 for 48 h and normalized by the correponding shControl cells. Results are shown as mean ± SD of four independent experiments; *p < 0.05, Student *t* test. **D.** Colonies containing viable cells were detected by MTT after 10 days of culture of shSTMN1 and normalized by the corresponding shControl cells. Colony images are representative of one experiment and the bar graphs show the mean ± SD of at least six independent experiments; ***p < 0.0001; Student *t* test. The assays were performed in the presence or not of ruxolitinib (100 and 300 nM) as indicated.

In order to investigate the function of Stathmin 1 in a JAK2^V617F^ cell line, HEL cells were stably transduced with lentiviral constructs encoding shRNA targeting *Stathmin 1* (shSTMN1) or a shRNA targeting a control sequence (shControl). After polyclonal cell selection with puromycin, the efficient Stathmin 1 silencing was verified by qPCR and Western blotting (Figure 2A). We next evaluated the effect of Stathmin 1 silencing on cell viability and proliferation, in the presence or not of the selective JAK1/2 inhibitor ruxolitinib. Stathmin 1 silencing significantly reduced cell viabilty compared to control cells, and had an additive effect with ruxolitinib treatment (*p* < 0.05, Figure 2B). Ki-67 analysis revealed that Stathmin 1 silencing significantly reduced cell proliferation, but did not have additive effects with ruxolitinib treatment on cell proliferation (Figure 2C). Regarding long-term proliferative potential, Stathmin 1 silencing significantly reduced the number of colonies (*p* < 0.05, Figure 2D). Since ruxolitinib treatment (100 nM and 300 nM) strongly decreased colony formation, no further effect of Stathmin 1 inhibition on colony numbers was observed (Figure 2D), except in very low dose of ruxolitinib (10 nM; Supplementary Figure 1).

**Figure 3:**
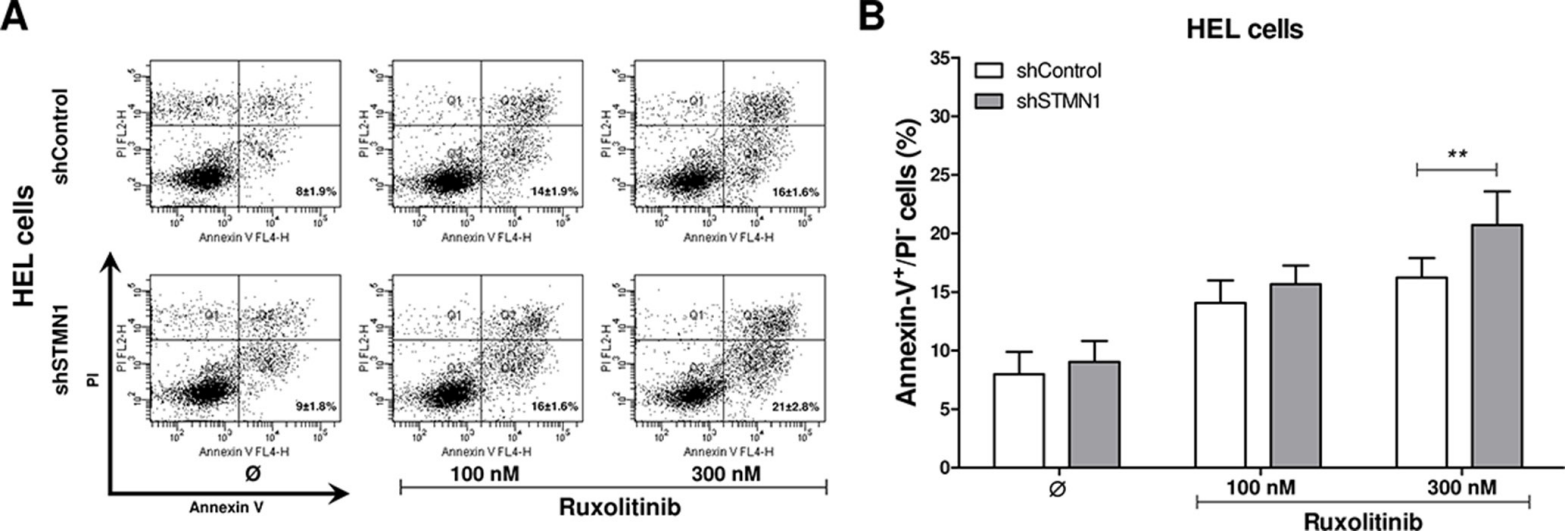
Stathmin 1 inhibition increases the pro-apoptotic effects of ruxolitinib treatment **A.** Apoptosis was detected by flow cytometry in HEL cells transduced with shControl and shSTMN1 using Annexin-V/PI staining method and a representative dot plot is illustrated. **B.** Bar graphs show the mean ± SD of six independent experiments; **p < 0.01; Student *t* test. The assays were performed in the presence or not of ruxolitinib (100 and 300 nM) as indicated.

We then investigated whether the Stathmin 1-silencing-induced decreased cell number was also due to increased apoptosis. Flow cytometry analysis revealed that Stathmin 1 silencing did not alter apoptosis in DMSO-treated HEL cells. However, Stathmin 1 silencing significantly increased apoptosis induced by ruxolitinib treatment at 300 nM (*p* < 0.01, Figure 3). Taken together, these results indicate that Stathmin 1 silencing alone decreases the proliferation and colony formation of HEL cells. When combined with ruxolitinib, Stathmin 1 silencing amplifies ruxolitinib-induced apoptosis in JAK2^V617F^ cells.

### Inhibition of JAK2/STAT3 signaling increases Stathmin 1 activity and microtubule instability in HEL cells

**Figure 4:**
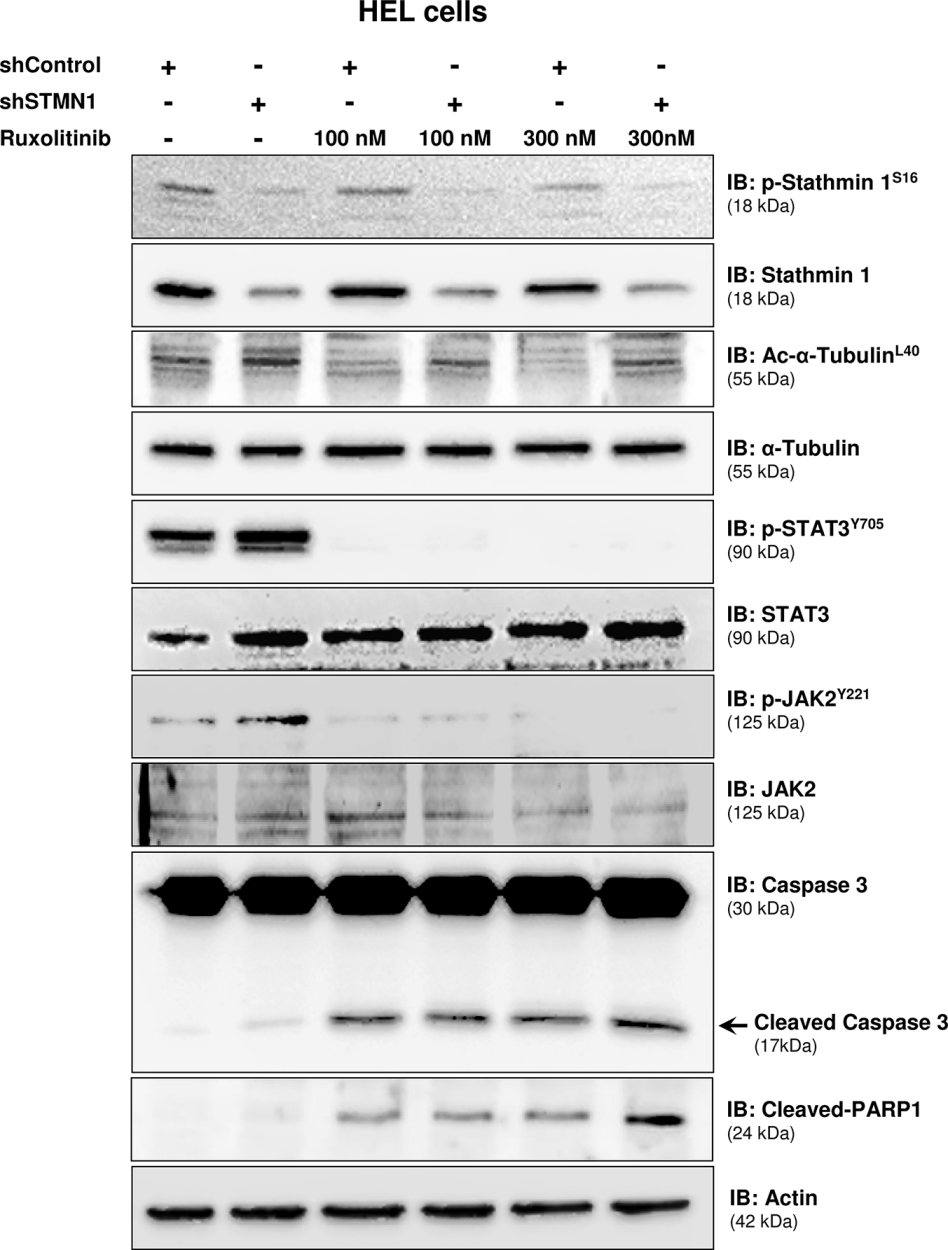
Stathmin 1 silencing prevents microtubule instability induced by ruxolitinib treatment Western blot analysis for p-Stathmin 1^S16^, Stathmin 1, alpha-tubulin acetylation (Ac-alpha-tubulin^L40^), p-STAT3^Y705^, p-JAK2^Y221^, caspase 3 (total and cleaved) and cleaved PARP1 levels in total cell extracts from shControl and shSTMN1 cells treated or not with ruxolitinib at 100 or 300 nM; membranes were reprobed with the antibody for detection of the respective total protein or actin, and developed with the ECL Western Blot Analysis System.

The effects of Stathmin 1 silencing, in combination or not with ruxolitinib treatment, on microtubule dynamics and apoptosis were assessed by the evaluation of alpha-tubulin acetylation and caspase 3/PARP1 cleavage, respectively. Immunoblotting analysis of shControl cells treated with ruxolitinib revealed decreased alpha-tubulin acetylation levels, indicating increased microtubule instability. In contrast, Stathmin 1 silencing increased acetyl-alpha-tubulin levels and prevented the loss of microtubule stability induced by ruxolitinib. As expected, ruxolitinib treatment reduced JAK2 and STAT3 phosphorylation levels in both shSTMN1 and shControl cells (Figure 4). In agreement with Annexin V staining results, the levels of cleaved caspase 3 and cleaved PARP1 increased upon ruxolitinib treatment, and higher levels of caspase 3/PARP1 cleavage were observed in shSTMN1 compared with shControl cells treated with 300 nM ruxolitinib (Figure 4). These results indicate that Stathmin 1 is involved in the microtubule instability during ruxolitinib treatment, possibly due to the downregulation of STAT3 activity.

### Paclitaxel-induced microtubule stability improves ruxolitinib response

**Figure 5:**
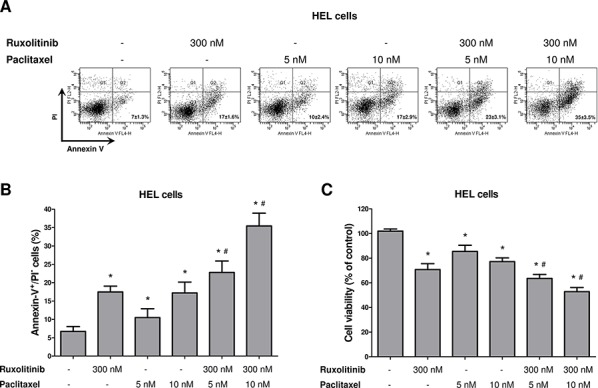
Paclitaxel-induced microtubule stability improves ruxolitinib response **A.** Apoptosis was detected by flow cytometry in HEL cells treated or not with paclitaxel (5 or 10 nM) and/or ruxolitinib (300 nM) using Annexin-V/PI staining method and a representative dot plot is illustrated. **B.** Bar graphs show the mean ± SD of six independent experiments; *p < 0.01 *vs*. untreated cells, ^#^*p* ≤ 0.001 *vs.* ruxolitinib or paclitaxel monotherapy at the corresponding dose; Student's *t* test. **C.** Cell viability was determined by MTT assay after 48 hours of incubation of HEL cells treated or not with paclitaxel (5 or 10 nM) and/or ruxolitinib (300 nM), and normalized by untreated HEL cells. Results are shown as mean ± SD of six independent experiments; *p ≤ 0.002 *vs*. untreated cells, #*p* ≤ 0.02 *vs.* ruxolitinib or paclitaxel monotherapy at the corresponding dose; Mann–Whitney test.

**Figure 6:**
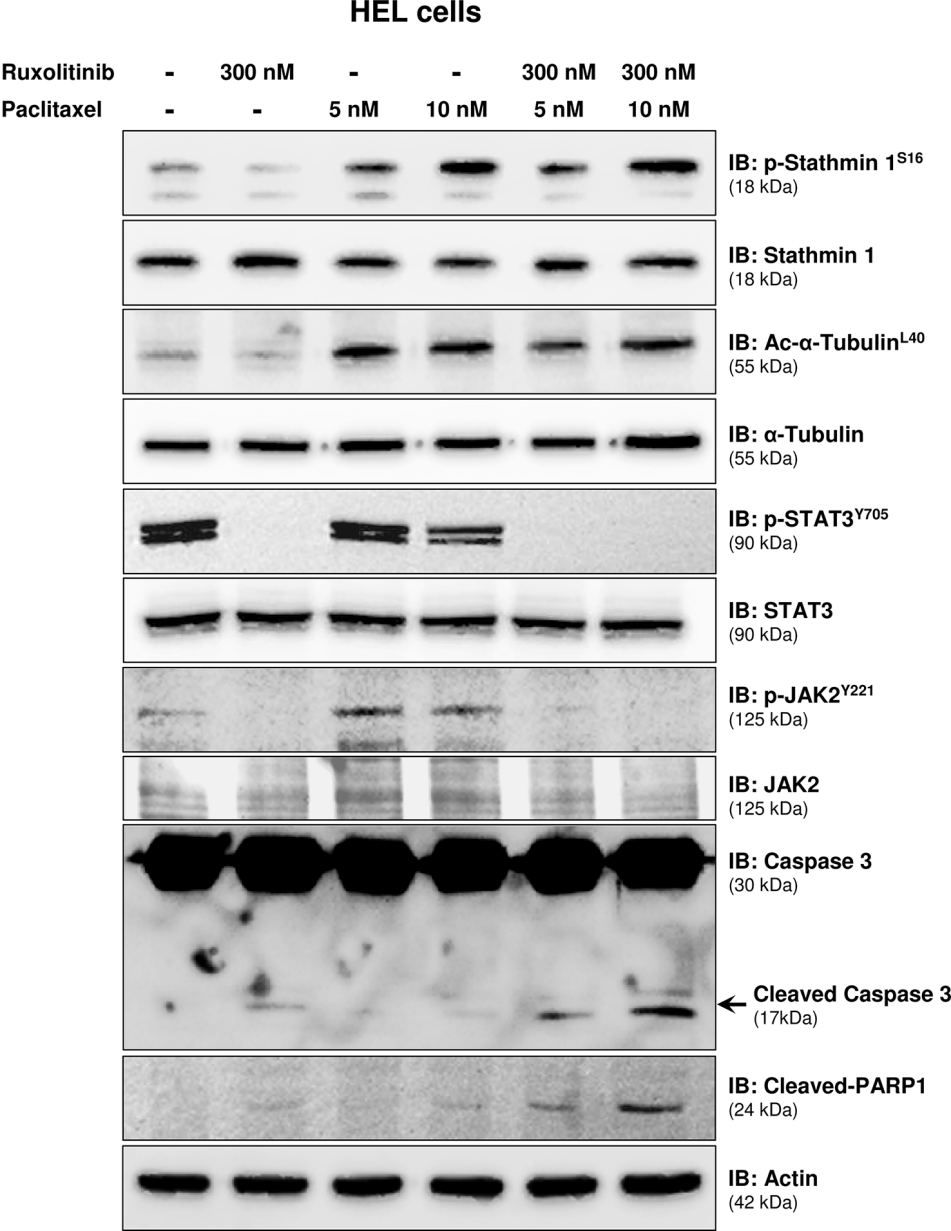
Paclitaxel treatment leads to Stathmin 1^S16^ phosphorylation and potentiates ruxolitinib-induced caspase 3/PARP1 cleavage Western blot analysis for p-Stathmin 1^S16^, alpha-tubulin acetylation (Ac-alpha-acetylation^L40^), p-STAT3^Y705^, p-JAK2^Y221^, caspase 3 (total and cleaved) and cleaved PARP1 levels in total cell extracts from HEL cells treated, or not, with paclitaxel (5 or 10 nM) and/or ruxolitinib (300 nM); membranes were reprobed with the antibody for the detection of the respective total protein or actin, and developed with the ECL Western Blot analysis system.

We next sought to evaluate whether pharmacological induction of microtubule stability by paclitaxel improved the response to ruxolitinib treatment, in a manner similar to the of Stathmin 1 silencing. Notably, paclitaxel combined with ruxolitinib treatment resulted in a greater reduction in cell viability and higher levels of apoptosis, compared with each treatment alone (Figure 5; *p* < 0.05). Western blot analysis indicated that paclitaxel was able to induce Stathmin 1 phosphorylation at serine 16 (an inhibitory site), overlapping the ruxolitinib effect and maintaining high levels of microtubule stability (Figure 6). Accordingly, paclitaxel plus ruxolitinib treatment resulted in increased levels of cleaved caspase 3 and cleaved PARP1 compared with paclitaxel or ruxolitinib alone (Figure 6).

### Stathmin 1 is highly expressed in primary myelofibrosis patients

**Figure 7:**
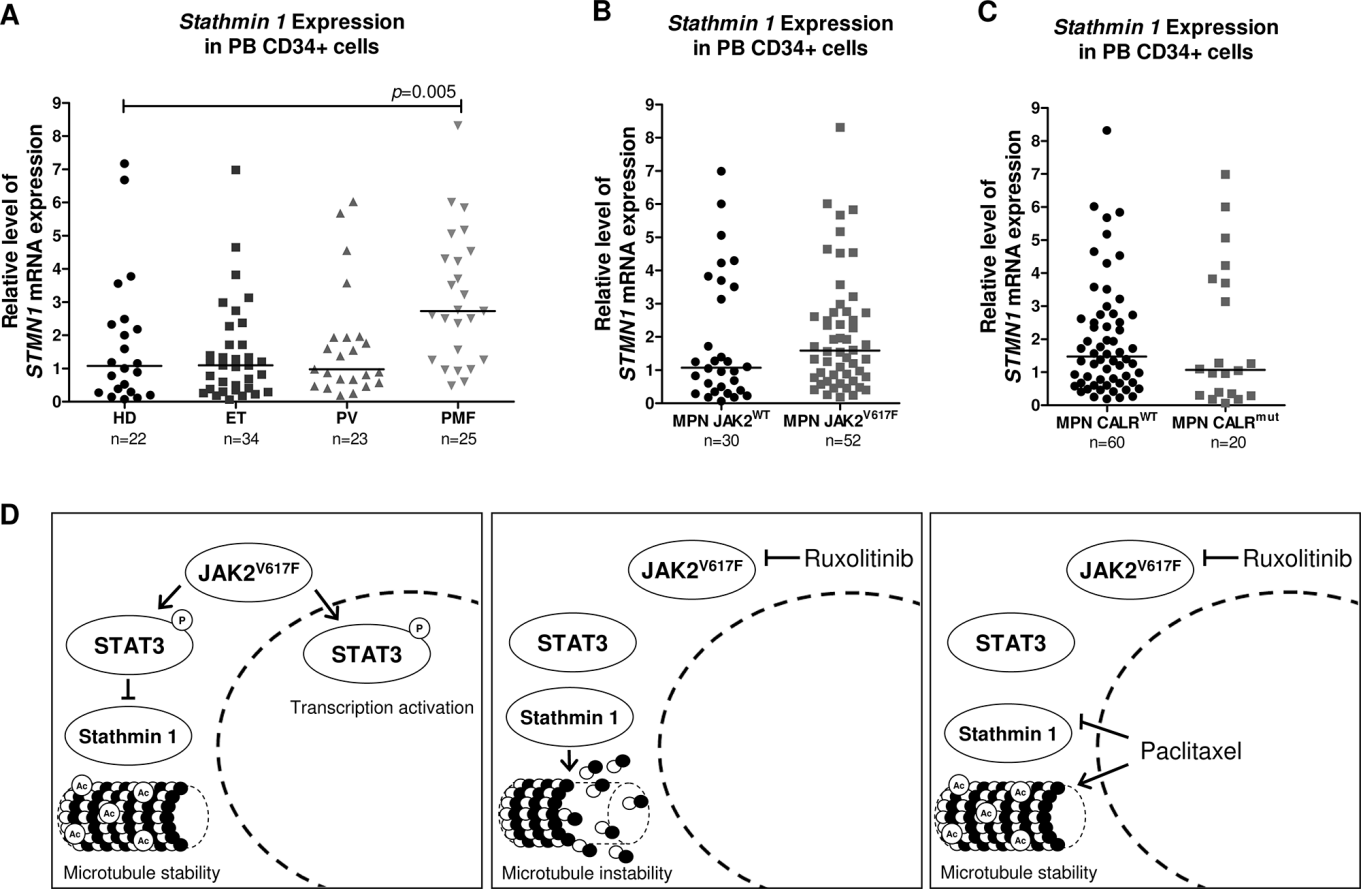
*Stathmin 1* mRNA levels in CD34^+^ cells from healthy donors and patients with myeloproliferative neoplasms **A.** qPCR analysis of *Stathmin 1* (*STMN1*) mRNA expression in peripheral blood (PB) CD34^+^ cells from healthy donors (HD), and from patients with a diagnosis of essential thrombocythemia (ET), polycythemia vera (PV) and primary myelofibrosis (PMF), and from myeloproliferative neoplasms (MPN) patients stratified by **B.** JAK2^V617F^ or **C.** CALR exon 9 mutational status. Horizontal lines indicate medians. The number of subjects and *p* values (Mann–Whitney test) are indicated in the graph. **D.** Potential model for Stathmin 1 function in HEL cells; the constitutive activation of JAK2 by the V617F mutation leads to STAT3 phosphorylation, Stathmin 1 inhibition and microtubule stability. Ruxolitinib treatment inhibits JAK2 and STAT3 activity, decreasing the Stathmin 1 and STAT3 association, which consequently releases Stathmin 1 and leads to microtubule instability. Paclitaxel treatment overcomes the effect of ruxolitinib treatment, inhibiting Stathmin 1 and increasing microtubule stability. Abbreviations: Ac: acetylation.

Aberrant Stathmin 1 expression has been described in several hematological malignancies, including lymphomas [8, 16], acute leukemias [7, 9, 17] and myelodysplastic syndromes [9, 18]. In addition, leukemia-related oncogenes, including BCR-ABL1 [19] and PML-RARα [20], have been associated with Stathmin 1 upregulation. Thus, we investigated *Stathmin 1* expression in PB CD34^+^ cells from healthy donors and patients with PV, ET and PMF and we also stratified the MPN patients according to the presence, or not, of JAK2^V617F^ and CALR mutations. *Stathmin 1* transcripts were significantly increased in PB CD34^+^ cells from PMF patients, compared with healthy donors (median 2.73 [range 0.49–8.32] *vs*. 1.08 [0.08–7.17], respectively, *p* = 0.005); however no significant difference in *Stathmin 1* expression was observed in PB CD34^+^ cells from ET (1.10 [range 0.07–6.98]) and PV (0.98 [0.18–6.02]) patients, compared with healthy donors (Figure 7A). *Stathmin* 1 expression did not differ in MPN patients when stratified according to JAK2 mutation status (JAK2^WT^ = 1.08 [range 0.07–6.99] *vs*. JAK2^V617F^ = 1.61 [range 0.19–8.32], *p* = 0.20) (Figure 7B) or CALR mutation status (CALR^WT^=1.48 [0.19–8.32] *vs*. CALR^MUT^ = 1.08 [range 0.07–6.99], *p* = 0.45) (Figure 7C).

## DISCUSSION

The relevance of Stathmin 1 in hematological malignancies has been well described in acute leukemia and lymphoma [7, 8, 9, 17, 21, 22]. However, studies addressing Stathmin 1 expression and function in MPN are still lacking. Using the JAK2^V617F^ HEL cell model, we observed that Stathmin 1 silencing reduced cell proliferation and clonal growth. These findings are in agreement with our previous results in U937 (myeloid leukemia cell line) and Namalwa (lymphoid leukemia cell line) cells [9], and in the BCR-ABL1 K562 cells, as reported by two independent groups [10, 11]. Importantly, in HEL cells, Stathmin 1 silencing presented an additional effect, futher reducting of cell viability and increasing the apoptosis induced by ruxolitinib, a selective JAK1/2 inhibitor approved by the FDA for the treatment of intermediate and high-risk PMF. In PMF patients, results from a phase III clinical trial demonstrated that ruxolitinib is well-tolerated, reduces inflammatory cytokines and splenomegaly, and ameliorates constitutional symptoms, but does not reverse bone marrow fibrosis [23, 24, 25], suggesting a requirement of additional therapeutic strategies. Thus, preclinical studies using combined drugs are emerging in an attempt to improve the response to JAK inhibitors [26, 27, 28, 29, 30].

Ruxolitinib inhibits the constitutional activation of the JAK/STAT pathway, including STAT3 [15]. The cross-talk between STAT3 and Stathmin 1 signaling has been previously described in other cell lines from mice (NSC-34 mouse motor neuron-like hybrid cells), rats (PC12 pheochromocytoma cells) [31] and humans (Hut78 T-lymphoma cells [13], and SGC7901 and MGC803 gastric cancer cells [14]). STAT3 is a nuclear transcriptional activator that also induces microtubule stability by the inactivation of Stathmin 1 in the cytoplasm [13, 14]. We then identified the cross-talk between STAT3 and Stathmin 1 signaling, also in HEL cells. Ruxolitinib downregulated STAT3 activity and STAT3/Stathmin 1 association, releasing and activating Stathmin 1 and increasing microtubule instability. Corroborating this hypothesis, microtubule stability was rescued by Stathmin 1 silencing during ruxolitinib treatment.

In order to verify whether microtubule stability might be involved in the improved response of Stathmin 1 silenced cells to ruxolitinib, paclitaxel was used. Paclitaxel is a microtubule-targeted chemotherapeutic drug that induces microtubule polymerization, cell-cycle block at the metaphase-anaphase transition and cell death, and is the first line treatment for specific solid tumors [32]. Interestingly, combined paclitaxel plus ruxolitinib treatment resulted in a significant reduction in cell viability and increased apoptosis, compared to monotherapy. In HEL cells, we then observed that paclitaxel treatment resulted in the same cell phenotype, as induced by Stathmin 1 silencing. Our results are summarized and illustrated in Figure 7D.

*Stathmin 1* expression was found to be upregulated in CD34^+^ cells from PMF patients. This finding can not yet be fully explained. In solid tumors, high Stathmin 1 expression correlates with tumor growth and progression [3]. Of note, Stathmin 1 expression is associated with the proliferative potential of early hematopoietic progenitors [4]. Evidence from *ex vivo* studies indicate that PMF CD34^+^ cells have a proliferative advantage, when compared to normal CD34^+^ cells [33]. Thus, we speculate that the high expression of Stathmin 1 in PB CD34^+^ PMF samples may contribute to the increased proliferative potential during early stages of cell differentiation. In contrast, in differentiated-MPN cells, Stathmin 1 inhibition and STAT3 activation may predominate. An important study recently found that Stat3 deletion increased hematopoietic stem cell compartments in Jak2^V617F^ knock-in mice [34], corroborating the relevance of STAT3 activation in differentiated cells. It should also be pointed out that gene expression analysis may not reflect the protein activity in primary MPN cells. Given the relevance of Stathmin 1 in HEL JAK2^V617F^ cells, herein identified, further studies to better elucidate the participation of Stathmin 1/STAT3 axis in primary samples from PMF patients will be of importance.

We then proposed that an undesired effect of ruxolitinib treatment may constitute Stathmin 1 activation and microtubule instability in JAK2^V617F^ cells. Pharmacologic inhibition of Stathmin 1, in association with ruxolitinib treatment, may be an interesting strategy for inducing apoptosis in JAK2^V617F^ cells. Our findings add new insights for Stathmin 1 involvement in the JAK2^V617F^ signaling pathway in MPN.

## MATERIALS AND METHODS

### Primary samples

CD34^+^ cells were obtained from a total of 22 peripheral blood (PB) samples collected from healthy donors (median age 44.5 years [range 37.3–51.1]) selected from the Blood Bank of the institution, and from 82 patients with MPN (median age 63.9 years [range 20.0–87.5]), including ET (*n* = 34), PV (*n* = 23) and PMF (*n* = 25) followed in the outpatient clinics of the University of Campinas. This study was approved by the Institutional and National Review Board in accordance to the Helsinki Declaration;. Patients were submitted to diagnosis evaluation according to the Word Health Organization 2008 criteria [35]. Seventy-six out of 82 patients were in regular use of hydroxyurea at the time of sampling, and none of the patients had received chemotherapy nor JAK1/2 inhibitor treatment before sampling. Among the patients, 52 were positive for JAK2^V617F^ mutation, 20 for CALR exon 9 *indel* mutation, 9 were double negative for both mutations, and 1 patient was JAK2 wild-type but had not been tested for CALR mutation. JAK2 and CALR mutations were investigated as previously described [36, 37].

### Cell culture and chemical reagents

The HEL cell line, which is known to harbor the JAK2^V617F^ mutation, was obtained from ATCC, Philadelphia, PA, USA. Cells were cultured in RPMI containing 10% fetal bovine serum (FBS) and glutamine with penicillin/streptomycin and amphotericin B, and maintained at 37°C, 5% CO_2_. Ruxolitinib, a selective JAK1/2 inhibitor, was obtained from Novartis Pharmaceuticals (Basel, Switzerland). Paclitaxel, a microtubule-stabilizing drug, was obtained from INTAS Pharmaceuticals (Ahmedabd, India).

### Quantitative PCR (qPCR) analysis

Quantitative PCR (qPCR) was performed with an ABI 7500 Sequence Detector System (Applied Biosystems, Foster City, CA, USA) with specific primers for *Stathmin 1* (forward: AGCCCTCGGTCAAAAGAATC; reverse: TTCAAGACCTCAGCTTCATGGG) [38] and *HPRT (hypoxanthine phosphoribosyltransferase 1*; forward: GAACGTCTTGCTCGAGATGTGA; reverse: TCCAGCAGGTCAGCAAAGAAT). The relative quantification value was calculated using the equation 2^−ΔΔCT^ [39]. A negative ‘No Template Control’ was included for each primer pair. The dissociation protocol was performed at the end of each run to check for non-specific amplification. Three replicas were run on the same plate for each sample.

### Immunoprecipitation and Western blot

Equal amounts of protein were used for total extracts or for immunoprecipitation with specific antibodies, followed by SDS-PAGE and Western blot analysis with the indicated antibodies (carried out using the ECL™ Western Blotting Analysis System; Amersham Pharmacia Biotech Ltd., Buckinghamshire, UK), as previously described. Antibodies against p-Stathmin 1 (p-OP18 S16, sc-12948-R), Stathmin 1 (OP18, sc-55531), alpha-tubulin (sc-5286), JAK2 (sc-294), STAT3 (sc-7179), PARP1 (sc-56197) and actin (sc-1616) were from Santa Cruz Biotechnology (Santa Cruz, CA, USA). Antibodies against p-JAK2 Y221 (#3774S), p-STAT3 Y705 (#9131S) and caspase 3 (#8G10) were from Cell Signaling Technology (Danvers, MA, USA). The antibody against acetyl-alpha-tubulin L40 (ab24610) was from Abcam (Cambridge, MA, USA).

### Confocal immunofluorescence microscopy

HEL cells treated or not with ruxolitinib (100 or 300 nM) for 48 hours, were attached on cover slips coated with poly-L-lisine (1 mg/mL), fixed with 4% paraformaldehyde, permeabilized with 0.5% Triton X-PBS and blocked with 3% bovine serum albumin (BSA) PBS. Cells were then incubated with anti-alpha-tubulin Alexa Fluor^®^ 488 conjugate (1:200 in 3% BSA PBS; eBioscience, San Diego, CA, USA) for 12 hours, and followed by three PBS washes. The slides were mounted in ProLong Gold Anti-Fade Mounting Medium with DAPI (Life Technologies, Carlsbad, CA, USA). Images were generated using a confocal laser-scanning microscope (LSM 510, Carl Zeiss, Welwyn Garden City, UK).

### Lentivirus transduction

HEL cells were transduced with lentivirus-mediated shRNA nonspecific control (sc-108080) or lentivirus-mediated shRNA targeting Stathmin 1 (containing three target-specific constructs; sc-36127-V) from Santa Cruz Biotechnology (Santa Cruz Biotechnology) and named shControl and shSTMN1 cells, respectively. Briefly, 2 ×10^5^ cells were transduced with lentivirus by spinoculation at multiplicity of infection equal to 1 and selected by 0.75 μg/mL puromycin.

### Methylthiazoletetrazolium (MTT) assay

Cell viability was measured by MTT assay. ShControl and shSTMN1 cells were serum-starved in 0.5% FBS for 12 hours. A total of 2.5 × 10^4^ cells per well were then cultured in a 96-well plate in RPMI 10% FBS in presence or not of ruxolitinib (100 and 300 nM). Paclitaxel (5 and 10 nM) was also used in the presence or not of ruxolitinib (300 nM). Next, 10 μL of a 5 mg/mL solution of MTT were added to the wells followed by incubation at 37°C for 4 hours. The reaction was stopped using 100 μL of 0.1N HCl in anhydrous isopropanol. Cell viability was evaluated by measuring the absorbance at 570 nm, using an automated plate reader. All conditions were tested in six replicates.

### Assessment of cell proliferation by Ki-67 staining

Cells were treated, or not, with different concentrations of ruxolitinib (100 or 300 nM) for 48 hours, fixed with 70% ethanol and stored at −20°C. Ki-67 staining was performed following the manufacturer's instructions (Ki-67 FITC clone B56; BD Bioscience, San Jose, CA, USA) and the mean of fluorescence intensity (M.F.I) was obtained by flow cytometry using a FACSCalibur (Becton Dickinson, San Jose, CA, USA). IgG isotype was used as negative control for each condition. Ten thousand events were acquired for each sample.

### Colony formation assay

Colony formation was carried out in semisolid methyl cellulose medium (1×10^3^ cell/mL; MethoCult 4230; StemCell Technologies Inc., Vancouver, BC, Canada). Colonies were detected after 10 days of culture by adding 1 mg/mL of MTT reagent and scored by Image J quantification software (U.S. National Institutes of Health, Bethesda, MD, USA).

### Assessment of apoptosis by Annexin V and PI staining

Cells were seeded on 24-well plates and treated or not with different concentrations of ruxolitinib (100 or 300 nM) and/or paclitaxel (5 or 10 nM) for 48 hours. Cells were then washed twice with ice cold PBS and resuspended in binding buffer containing 1 μg/mL PI and 1 μg/mL APC labeled Annexin-V. All specimens were analyzed by flow cytometry (FACSCalibur; Becton Dickinson) after incubation for 15 minutes at room temperature in a light-protected area. Ten thousand events were acquired for each sample.

### Statistical analysis

Statistical analyses were performed using GraphPad Instat 5 (GraphPad Software, Inc., San. Diego, CA, USA). For comparisons, Student's *t*-test or Mann–Whitney test were used for measured factors, as appropriate. A *p* value <0.05 was considered as statistically significant.

## SUPPLEMENTARY FIGURE


