# Supplementary material for: Stathmin 1 inhibition amplifies ruxolitinib-induced apoptosis in JAK2V617F cells
Source: Oncotarget. 2015 Aug 17;6(30):29573–84. doi: 10.18632/oncotarget.4998 (PMC4745747; doi:10.18632/oncotarget.4998)
Supplement: Supplementary file 1 [file oncotarget-06-29573-s001.pdf]

SUPPLEMENTARY FIGURE

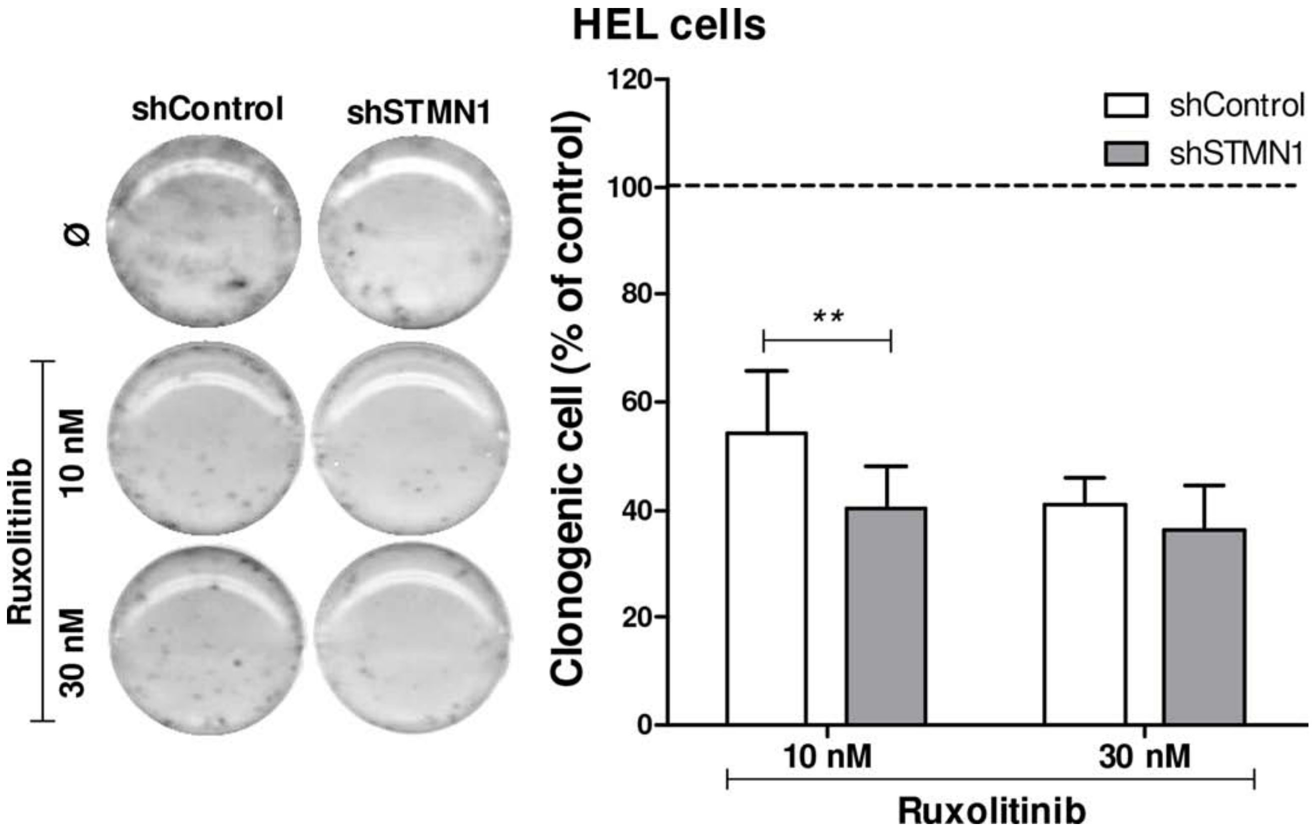

Supplementary Figure S1: Stathmin 1 silencing reduces clonogenicity in HEL cells. Colonies containing viable cells were detected by MTT after 10 days of culture of shSTMN1 and normalized by the corresponding shControl cells. Colony images are representative of one experiment and the bar graphs show the mean ± SD of six independent experiments; $^{**}p < 0.001$; *Student t* test. Dotted line represents the mean of untreated-shControl HEL cells. The assays were performed in the presence or not of ruxolitinib (10 and 30 nM) as indicated. ShControl and shSTMN1 untreated-HEL cells data are shown in Figure 2D.
